# Chronic health conditions and work-related stress in older adults participating in the Dutch workforce

**DOI:** 10.1007/s10433-020-00554-x

**Published:** 2020-02-14

**Authors:** Miriam Mutambudzi, Kene Henkens

**Affiliations:** 1 MRC/CSO Social and Public Health Sciences Unit, Institute of Health and Wellbeing, University of Glasgow, Glasgow, G2 3AX UK; 2 Netherlands Interdisciplinary Demographic Institute (NIDI-KNAW), The Hague, The Netherlands; 3 University Medical Center Groningen (UMCG), University of Groningen, Groningen, The Netherlands; 4 Department of Sociology, University of Amsterdam, Amsterdam, The Netherlands

**Keywords:** Ageing workforce, Chronic health conditions, Emotional demands, Physical demands, Work-related stress

## Abstract

The proportion of workers with chronic health conditions (CHCs) will increase over the years as pension reform is increasing the age of retirement in many European countries. This will increase the percentage of older adults with CHCs performing highly demanding work. This study sought to examine the association between common CHCs [cardiovascular disease (CVD), diabetes, arthritis, respiratory and sleep disorders] and three domains of work stress in older Dutch workers. This study used data from the first wave of the NIDI Pension Panel Study for working adults aged 60–65 years (*n* = 6793). Logistic regression models examined the strength of association between CHCs and (1) general work stress, (2) emotional, and (3) physical demands. All five CHC were independently associated with one or more domains of stress. After including all CHCs in the model, CVD, sleep disorders, and arthritis were significantly associated with general stress. Respiratory disorders, sleep disorders, and arthritis were significantly associated with physical demands. Diabetes (1.25, 95% CI 1.01–1.53), sleep disorders (1.99, 95% CI 1.72–2.31), and arthritis (1.18, 95% CI 1.06–1.31) were significantly associated with emotional demands. Our findings demonstrate that work stress is associated with prevalent CHCs, and these conditions are differentially associated with several domains of work stress in adults approaching retirement. More research is needed to understand the causal relationship between CHCs and work stress. Such research may provide insights for effective workplace and public health interventions to ensure that older workers remain physically and mentally healthy, and productive through their working years.

## Introduction

The prevalence of independent and comorbid chronic health conditions (CHCs) increases with age (Koolhaas et al. 2013). Approximately 66% of the Dutch population aged 50–65 have at least one chronic condition, and common risk factors for CHCs that include high BMI, smoking and reduced physical activity have seen increasing trends in older Dutch adults over the years (Rezayatmand et al. 2015; Koopman et al. 2016). In addition to their association with disability and mortality, CHCs such as cardiovascular disease (CVD), diabetes, sleep disorders, rheumatic diseases and respiratory disorders can negatively impact work life (Graaf et al. 2012; Koolhaas et al. 2013). High prevalence of these conditions in older workers, coupled with extensions to the work life due to pension reform in many European countries, will result in larger proportions of workers with CHCs, which can affect labour force participation and productivity (Koolhaas et al. 2013; Mutambudzi et al. 2019a; Oude Mulders 2019). Approximately 20% of employees within the EU have a long-standing illness or health problem, and one in three mid-old age employees in the Netherlands reports a CHC, with 30% experiencing job difficulties associated with their disease (Detaille et al. 2009; Koolhaas et al. 2012; European Foundation for the Improvement of Living and Working Conditions 2014).

Workers with CHCs report greater exposure to work stress and perceive their work as more mentally strenuous relative to their healthier colleagues (Koolhaas et al. 2012; European Foundation for the Improvement of Living and Working Conditions 2014). Approximately one quarter of employed adults in the EU reports job strain, and annual costs of work-related stress factors are estimated to be around €4–€6 billion in the Netherlands, almost 3% of the country’s GDP (Hassard 2014). Work stress is not only associated with subsequent poor health, but is known to adversely impact job satisfaction, productivity, quality of life, and work life balance, as well as increased incidence of sickness absence, and premature exit from the workforce through early retirement or disability pension (Ganster and Rosen 2013).

Research assessing work stress as an outcome of adverse health is lacking. Previous studies primarily reported that stressful work environments are explanatory factors for increased risk of onset, or exacerbation of CHCs (Koolhaas et al. 2013; Mutambudzi and Javed 2016). Some scholars have postulated that this association is bidirectional, with several suggesting that work stress levels increase after onset of CVD, diabetes, and sleep disorders (Li et al. 2015; Van Laethem et al. 2015). Li and colleagues measured work stress before and after onset of CVD and reported that work stress increased after workers diagnosed with CVD returned to work (Li et al. 2015). Another study which examined the bidirectional relationship between sleep and stress found that poor sleep quality was associated with increased work stress the following year, while work stress was not associated with subsequent decreased sleep quality. In addition to studies that examined a bidirectional association, some studies have demonstrated that CHCs such as rheumatic diseases and diabetes can confound an employee’s ability to optimally perform work duties, thereby increasing risk of experiencing poor mental and psychosocial outcomes, due to concerns of how their compromised work ability may impact their financial or job security and quality of life (Graaf et al. 2012; Mutambudzi et al. 2019a; Vanajan et al. 2019).

There are several mechanisms through which poor chronic health may increase risk of work stress. CVD, diabetes, rheumatoid diseases, and respiratory disorders are associated with reduced health-related work performance and poor quality of working life after disease onset (Graaf et al. 2012; Mutambudzi et al. 2019a). Existing health problems, their treatment side effects and poor disease management may result in chronic fatigue, poor concentration, impaired functioning, increased presentism or absenteeism, and decreased productivity (Graaf et al. 2012; de Jong et al. 2015; Mutambudzi et al. 2019a). These factors may induce stress through impacting promotion and salary increment opportunities, prompting changes to the work role, and potential demotions or job loss (de Jong et al. 2015). Similarly, lack of quality sleep can negatively impact work performance, attention to tasks and decision-making factors that may increase workload and work-related stress (Van Laethem et al. 2015). In addition, discrimination and prejudice in the workplace which have been associated with emotional stress disproportionally affect workers with chronic diseases (Okechukwu et al. 2014). Research indicates that the manifestation of general “non-specific” work stress, physical demands, and qualitative demands including emotional stress may be differentially patterned by varying factors (Rivera-Torres et al. 2013) and influence different aspects of human functioning, making it imperative to assess these domains separately (de Jonge et al. 2010). Of note, muscle mass decreases with age, making tasks that require physical exertion challenging (Sundstrup et al. 2016), while chronic exposure to emotionally charged interactions or events, and work that involves catering to the emotional needs of patients or clients can result in chronic fatigue and burnout (Scheibe et al. 2015). As the structure of the population changes due to longer life expectancies and extended work lives, the proportion of older adults in jobs with high emotional and physical demands is increasing (Scheibe et al. 2015; Oude Mulders 2019). There is, however, limited knowledge of how CHCs highly prevalent in older adults may be explanatory factors for these work demands.

Despite plausible pathways of the association between poor health and greater risk of work stress, most studies continue to treat work stress primarily as a predictor rather than an outcome of adverse health. Less is therefore known of the independent and comorbid effects of CHCs on risk of work-related stress in the older workforce. Studies assessing how varied CHCs may be differentially associated with different domains of stress in adults approaching retirement are scarce, but vital, given that CHCs have a high prevalence in this subpopulation (Koolhaas et al. 2013) and the increasing age of retirement. Further, analyses on work stress as an outcome of poor health will contribute to the literature and discussion on the bidirectional relationship between these factors when taken along with other similar studies, and may provide insights into appropriate workplace and public health interventions, to ensure older workers are healthy and able to remain productive.

Given the gaps in the literature, the goal of this study was to examine whether common CHCs in older adults were associated with work-related stress. Specifically, we assessed if independent and comorbid CVD, respiratory disorders, diabetes, sleep disorders and arthritis were associated with (1) general stress, (2) emotional demands, and (3) physical demands, while accounting for relevant factors. We chose to focus our analyses on these five highly prevalent conditions which are known to negatively impact health, well-being, and quality of life of older adults. Sleep disorders reportedly increase with age and approximately 45% of older adults report insomnia, while obstructive sleep apnoea affects 25–35% of adults over the age of 60 years (Alessi and Vitiello 2015; Yaremchuk 2018). Arthritis is reported to most frequently co-occur with other health conditions and is the most common cause of disability (Hootman et al. 2012; Garin et al. 2016). According to a position statement on behalf of the International Association of Gerontology and Geriatrics, the European Diabetes Working Party for Older People, and the International Task Force of Experts in Diabetes, approximately one in five older adults has a diabetes diagnosis (Sinclair et al. 2012). Diabetes complications negatively impact quality of life and may lead to disability and premature mortality (Holman et al. 2013; Mutambudzi et al. 2019b). Chronic diseases account for 90% of deaths in the Netherlands, and CVD, chronic respiratory diseases, and diabetes jointly account for approximately 35% of these deaths (World Health Organization 2014).

## Methodology

### Dataset description

This study used data from the first wave of the NIDI Pension Panel Study collected in 2015 (Henkens et al. 2017). Using a stratified design, a sample of organizations were selected from three large Dutch pension funds, after which workers aged 60–65, working 12 or more hours per week were randomly sampled from the selected organizations. Questionnaires were mailed to 15,470 potential respondents’ home, who also had the option to complete the questionnaire online. Of the participants invited to participate, 6793 completed the questionnaire, representing a 44% response rate at baseline.

### Variables of interest

The outcome of interest was work-related stress. Participants were asked whether they experienced (1) “stress,” (2) emotional demands, and (3) physical demands in their work. The response options very, fairly, a little, or no were dummy coded (very, fairly = 1), no (a little, no = 0). These measures were adopted from the Study on Transitions in Employment, Ability and Motivation (STREAM) survey (Van Vegchel et al. 2004). We defined general “stress” as non-specific stress. Emotional demands refers to work that requires sustained emotional effort in catering to the needs of patients or clients (Van Vegchel et al. 2004; Scheibe et al. 2015). Physical demands refers to work that requires physical exertion and involves lifting, carrying, pushing or pulling heavy loads, bending twisting, crouching, kneeling, standing for long periods of time, and repetitive movements with hands or arms (Sterud 2014; Andersen et al. 2016).

The independent variables of interest were CHCs which were ascertained from responses to a question asking participants whether a doctor had diagnosed one or more of a list of long-standing diseases. Each response was dichotomized (yes/no). For the purposes of this study, we focused on five common CHCs among older adults that are highly prevalent and associated with poor health, increased risk of morbidity, disability, and mortality. These included (1) CVD, (2) diabetes, (3) arthritis, (4) sleeping disorders, and (5) respiratory disorders.

### Covariates

Based on the previous literature demonstrating effects on work-related stress, additional variables of interest controlled for in the analyses included gender (male, female), partner/marital status (single, have a partner), age respondent entered the work force (< 18 years, 18–25 years, > 25 years), work hours per week, schedule and location flexibility (flexible, not flexible), International Socio-Economic Index (ISEI-08), occupational sector (government, education, construction, health care), occupational group (blue [manual work]/while collar), current smoker (yes/no), alcohol use more than twice a week (yes/no) and paying attention to body weight (yes/no). The International Socio-Economic Index of Occupational Status (ISEI-08) is a continuous variable indicating standardized scores that measure occupational status. This measure takes into account education and earnings, with higher ISEI-08 scores indicating higher occupational status.

### Statistical analysis

Approximately 85.5% of the cases had complete data, and item non-response ranged from approximately 0.01 to 5.1% for each question. The outcomes of interest contained 3–5% missing data. As performing complete case analyses may result in biased estimates and inflated standard errors (SE), we conducted diagnostic tests using *t* tests and Chi-squared tests to assess missing patterns in the variables of interest. We observed some social patterning in missingness between some variables; for example, approximately 78.1% of those who had missing data points on work hours did not have location flexibility. The systematic relationship between the propensity of missing values and observed data indicated that data were missing at random for the variables of interest. To account for the missing data, we used multiple imputation to estimate probable value ranges for incomplete observations. Multiple imputation generates a set of replacements for the missing values based on plausible models for the data which produces multiple completed datasets for analysis (Rubin 1987). We used multiple imputation by chained equations (MICE) which allows for each variable containing missing data to be regressed on all other variables (Hurtado et al. 2012). This method of accounting for missing data has been demonstrated to produce asymptotically unbiased estimates and standard errors (Hurtado et al. 2012). Twenty imputed datasets were generated and thereafter assessed according to multiple imputation procedures described by Rubin (1987).

Sample characteristics were summarized using frequencies and means. Logistic regression models for which odds ratios (OR) and 95% CIs were reported, examined the strength of association between CHCs and (1) general work stress, (2) emotional demands, and (3) physical demands. Three models fully adjusted for all the covariates were estimated. The first independently assessed the relationship between each health condition individually and each of the work stress outcomes. The second model included all five health conditions in the model, which in so doing accounted for comorbid CHC. We also created a multimorbidity variable with three distinct categories representing no disease, one condition only, and two or more of the five conditions of interest and used it to estimate a third model assessing multimorbidity risk of each stress domain. All analyses were performed using Stata14 MP Software (Stata, College Station, TX).

## Results

Table 1 presents the participant baseline characteristics. The average age at baseline was 62 (SD 1.6). Participants were predominantly male (53.9), 49% started working between the ages of 18–25, and the average hours worked per week were 31.8 (SD 8.96). Prevalence of CVD, respiratory disorders, diabetes, arthritis, and sleep disorders were 12.5%, 8.4%, 6.4%, 45.5%, and 15.7%, respectively.

**Table 1:** Baseline characteristics

Variable	%
Age (mean, SD)	62.0, 1.6
Gender	
Male	53.9
Female	46.1
Partner	
Partner	81.0
No partner	19.1
Age started working	
< 18 years	43.5
18–25 years	49.1
> 25 years	7.4
Work hours per week (mean, SD)	31.8, 9.0
Standardized ISEI08 (mean, SD)	− 0.03, 0.95
Schedule flexibility	
Yes	53.7
No	46.4
Location flexibility	
Yes	22.2
No	77.8
Sector	
Government	27.1
Education	22.3
Construction	20.1
Health care	13.6
Welfare	16.9
Social support score (mean, SD)	2.7, 0.3
Blue/white collar	
White collar	77.4
Blue collar	22.6
Smoking	
Yes	15.8
No	84.3
Alcohol	
Yes	20.6
No	79.4
Pay attention to body weight	
Yes	83.6
No	16.4
Chronic health conditions prevalence	
CVD	12.5
Respiratory	8.4
Diabetes	6.4
Arthritis	45.5
Sleep disorders	15.7

Table 2 presents the results of the independent associations between each CHC and work-related stress. CVD (1.27, 95% CI 1.09–1.49), respiratory disorders (1.29, 95% CI 1.06–1.57), sleep disorders (2.29, 95% CI 1.96–2.69), and arthritis (1.28, 95% CI 1.15–1.42) were independently associated with an increased risk of general stress. Respiratory disorders, sleep disorders, and arthritis were also significantly associated with a 63% increased risk of reporting high physical demands. All five CHCs were independently associated emotional demands. Overall respiratory and sleep disorders and arthritis were associated with all three stress variables, and the strongest associations were observed between sleep disorders and general stress.

**Table 2:** Logistic regression results for the independent association between each chronic health condition and work-related stress

	General stress			Physical demands			Emotional demands		
	OR	95% CI		OR	95% CI		OR	95% CI	
CVD	**1.27**	**1.09**	**1.49**	1.02	0.84	1.22	**1.18**	**1.01**	**1.38**
Respiratory disorders	**1.29**	**1.06**	**1.57**	**1.63**	**1.31**	**2.02**	**1.24**	**1.03**	**1.49**
Diabetes	1.11	0.91	1.36	1.00	0.78	1.29	**1.31**	**1.06**	**1.60**
Sleep disorders	**2.29**	**1.96**	**2.69**	**1.63**	**1.38**	**1.93**	**2.08**	**1.80**	**2.41**
Arthritis	**1.28**	**1.15**	**1.42**	**1.63**	**1.44**	**1.86**	**1.27**	**1.15**	**1.41**

Fully adjusted for gender, partner, age started working, work hours per week, schedule flexibility, location flexibility, ISEI08, occupational sector, blue/white collar occupation, smoking, alcohol, and pay attention to body weight. Bold font indicates p < 0.05

The independent associations of each of the chronic health conditions were modelled separately and controlled for the above-mentioned covariates

After including all CHCs in the fully adjusted model (Table 3), CVD, sleep disorders, and arthritis remained significantly associated with general stress. The association between respiratory disorders and general stress, however, was attenuated and lost statistical significance. Respiratory disorders, sleep disorders, and arthritis remained significantly associated with physical demands. Sleep disorders (1.98, 95% CI 1.71–2.30) and arthritis (1.18, 95% CI 1.06–1.32) remained significantly associated with emotional demands.

**Table 3:** Logistic regression results for the association between all chronic health conditions and work-related stress

	General stress			Physical demands			Emotional demands		
	OR	95% CI		OR	95% CI		OR	95% CI	
CVD	**1.19**	**1.01**	**1.39**	0.95	0.78	1.15	1.09	0.93	1.28
Respiratory disorders	1.19	0.97	1.44	**1.51**	**1.21**	**1.87**	1.14	0.95	1.37
Diabetes	1.02	0.83	1.26	0.95	0.74	1.22	1.21	0.99	1.49
Sleep disorders	**2.18**	**1.86**	**2.56**	**1.49**	**1.26**	**1.76**	**1.98**	**1.71**	**2.30**
Arthritis	**1.18**	**1.06**	**1.31**	**1.56**	**1.37**	**1.77**	**1.18**	**1.06**	**1.32**
Gender (ref: male)	1.02	0.89	1.18	**1.28**	**1.08**	**1.54**	0.96	0.83	1.10
Partner (ref: married/partner)	0.96	0.83	1.10	0.95	0.80	1.12	0.93	0.81	1.08
ISEI08	**1.49**	**1.35**	**1.64**	0.68	0.60	0.76	**1.41**	**1.28**	**1.56**
Sector (ref: government)									
Education	**1.36**	**1.15**	**1.60**	**2.66**	**2.14**	**3.31**	**2.12**	**1.80**	**2.49**
Construction	**1.44**	**1.21**	**1.70**	**1.99**	**1.62**	**2.46**	1.11	0.93	1.33
Health care	**1.31**	**1.08**	**1.58**	**4.11**	**3.26**	**5.17**	**2.65**	**2.19**	**3.19**
Welfare	**1.46**	**1.24**	**1.73**	**2.44**	**1.95**	**3.05**	**2.18**	**1.84**	**2.58**
Age started working (ref > 25)									
< 18 years	**1.34**	**1.08**	**1.65**	**1.64**	**1.23**	**2.19**	**1.53**	**1.23**	**1.90**
18–25 years	**1.34**	**1.09**	**1.64**	**1.10**	**0.83**	**1.47**	**1.31**	**1.06**	**1.61**
Work hours per week	**1.03**	**1.02**	**1.04**	1.00	0.99	1.01	**1.02**	**1.01**	**1.03**
Schedule flexibility (ref: yes)	**1.78**	**1.59**	**1.99**	**1.88**	**1.65**	**2.15**	**1.66**	**1.49**	**1.86**
Location flexibility (ref: yes)	0.92	0.80	1.06	**2.31**	**1.88**	**2.85**	1.15	1.00	1.32
Smoking (ref: no)	0.95	0.81	1.11	**1.16**	**0.96**	**1.40**	1.07	0.92	1.26
Alcohol (ref: no)	0.98	0.86	1.13	0.90	0.75	1.06	1.07	0.94	1.23
Pay attension to body weight (ref: no)	1.01	0.87	1.17	1.07	0.90	1.28	1.05	0.91	1.22
Blue/white collar (ref: white collar)	0.88	0.71	1.09	**4.38**	**3.42**	**5.61**	0.90	0.72	1.11

Fully adjusted for gender, partner, age started working, work hours per week, schedule flexibility, location flexibility, ISEI08, occupational sector, blue/white collar occupation, smoking, alcohol, and pay attention to body weight. Bold font indicates p < 0.05

Some covariates were significantly associated with the three domains of stress in our fully adjusted models. Of particular interest were the associations with physical demands. Relative to men, women had a 28% increased risk of physical demands (95% CI 1.08–1.54). Further, as ISEI08 increased, i.e., as occupational status increased, general stress and emotional demands also increased; however, an inverse association was observed with physical demands. We also observed that in comparison with the government sector, all other industrial sectors had greater risk of the three domains of stress, with health-care workers exhibiting a fourfold increased risk of physical demands (95% CI 3.26–5.17). Similarly, blue-collar workers had an over fourfold increased risk of physical demands (95% CI 3.42–5.61). Lack of schedule flexibility increased risk of all three domains of stress, while lack of location flexibility increased risk of physical demands.

Table 4 presents results of the logistic regression for the association between multimorbid CHCs and the three domains of stress. Relative to having no CHC, having one condition was associated with a significant 26–51% increased risk of all three domains of stress. The presence of two or more CHCs was associated with a twofold increased risk in general stress (95% CI 1.81–2.44) and physical demands (95% CI 1.79–2.50), and a 96% increased risk of emotional demands (95% CI 1.70–2.26). Similar to findings reported in Table 3, strong associations were also observed between physical demands, health-care work and blue-collar work.

**Table 4:** Logistic regression results for the association between multimorbid chronic health conditions and work-related stress

	General stress			Physical demands			Emotional demands		
	OR	95% CI		OR	95% CI		OR	95% CI	
Multimorbidity (ref: 0)									
1 CHC	**1.26**	**1.12**	**1.41**	**1.51**	**1.31**	**1.74**	**1.34**	**1.19**	**1.50**
2 or more CHCs	**2.10**	**1.81**	**2.44**	**2.11**	**1.79**	**2.50**	**1.96**	**1.70**	**2.26**
Gender (ref: male)	1.03	0.89	1.18	1.31	1.10	1.56	0.96	0.84	1.10
Partner (ref: married/partner)	0.96	0.84	1.11	0.94	0.80	1.11	0.94	0.81	1.08
ISEI08	**1.50**	**1.37**	**1.66**	**0.68**	**0.60**	**0.77**	**1.42**	**1.29**	**1.56**
Sector (ref: government)									
Education	**1.35**	**1.15**	**1.59**	**2.68**	**2.16**	**3.33**	**2.11**	**1.79**	**2.48**
Construction	**1.42**	**1.20**	**1.69**	**2.02**	**1.64**	**2.49**	1.10	0.92	1.31
Health care	**1.32**	**1.10**	**1.60**	**4.14**	**3.29**	**5.22**	**2.66**	**2.21**	**3.22**
Welfare	**1.47**	**1.25**	**1.74**	**2.45**	**1.96**	**3.05**	**2.18**	**1.85**	**2.58**
Age started working (ref > 25)									
< 18 years	**1.33**	**1.08**	**1.65**	**1.64**	**1.23**	**2.18**	**1.52**	**1.23**	**1.90**
18–25 years	**1.34**	**1.10**	**1.64**	1.09	0.82	1.45	**1.31**	**1.06**	**1.61**
Work hours per week	**1.03**	**1.02**	**1.03**	1.00	0.99	1.01	**1.02**	**1.01**	**1.03**
Schedule flexibility (ref: yes)	**1.79**	**1.60**	**2.00**	**1.88**	**1.65**	**2.15**	**1.67**	**1.49**	**1.86**
Location flexibility (ref: yes)	0.93	0.81	1.06	**2.33**	**1.89**	**2.87**	1.15	1.00	1.32
Smoking (ref: no)	0.95	0.82	1.11	1.17	0.98	1.41	1.08	0.92	1.26
Alcohol (ref: no)	0.98	0.86	1.13	0.90	0.76	1.07	1.07	0.93	1.22
Pay attention to body weight (ref: no)	1.01	0.87	1.17	1.06	0.89	1.26	1.05	0.91	1.21
Blue/white collar (ref: white collar)	0.88	0.71	1.09	**4.34**	**3.39**	**5.56**	0.90	0.72	1.11

Fully adjusted for gender, partner, age started working, work hours per week, schedule flexibility, location flexibility, ISEI08, occupational sector blue/white collar occupation, smoking, alcohol, and pay attention to body weight. Bold font indicates p < 0.05

## Discussion

Our study sought to assess whether highly prevalent CHCs were associated with general stress, physical demands, and emotional demands. Overall, our results indicated that sleep disorders, respiratory disorders, and arthritis were significantly associated with all three stress domains. Studies assessing associations between these conditions as explanatory factors and stress as an outcome are scarce; however, several studies have suggested a reciprocal and reverse association between sleep and work stress (Van Laethem et al. 2015; Johannessen and Sterud 2017). Compromised sleep may lead to mental and physical fatigue, which may impair performance and adherence to safety guidelines, and alter perceptions of the work environment (Van Laethem et al. 2015; Johannessen and Sterud 2017). Studies have also indicated that patients with rheumatoid arthritis face challenges in the work place due to fatigue, poor range of motion, and pain which negatively impact their work ability, results in fear, anxiety, feelings of guilt and inadequacy, and fear of financial strain in case of job loss (Lacaille et al. 2007). There is also evidence from the literature that respiratory conditions such as COPD become incapacitating with progression and severity and can decrease ability to carry out tasks or engage in physically strenuous activities (Carter et al. 1994).

We found significant associations between independent CVD and general demands, and diabetes and emotional demands; however, both conditions were not associated with physical demands in the model that included all five CHC. While collinearity cannot be ruled out, it is also possible there was unaccounted selection bias in our data, due to the healthy worker survival effect (HWSE). The HWSE is characterized by self-selection out of jobs or the labour force market, by workers with poorer health, leaving workers who are generally healthier in the workplace (Pearce et al. 2007). These diseases have multiple daily self-management activities such as blood glucose monitoring and insulin administration (Ruston et al. 2013), which may be difficult to carry out in the work environment, particularly for workers with high occupational physical activity, thereby prompting premature exit from the workforce. An alternative explanation is that workers with self-efficacy and in supportive work environments are better able to manage their disease. Good disease self-management can decrease risk of adverse incidents such as rheumatic flare-ups or hypoglycaemia which impact work productivity, consequently leading to anxiety and stress (Brod et al. 2011; Verstappen 2015; Mutambudzi et al. 2019a).

Through our findings, we demonstrate that work stress may be a consequence of prevalent health conditions and that these conditions are differentially associated with several dimensions of work stress in adults approaching retirement. The average age of the workforce is steadily rising in industrialized countries, and thus, jobs with high physical and emotional demands are increasingly carried out by older employees (Scheibe et al. 2015). Our findings also alluded to different psychosocial and physical risk factors by sector. We observed that working in the government sector was protective of experiencing the three domains of stress, when compared to working in the other sectors. Relative to the government sector, the health-care sector had particularly strong associations with physical demands (OR 4.11). The finding of high physical demands among health-care workers is corroborated in the literature (Trinkoff et al. 2003; Koohpayehzadeh et al. 2016; Merkus et al. 2019), with evidence that older health-care workers are more vulnerable and likely to experience higher demands (Merkus et al. 2019). An equally high level of association was also observed between blue-collar work and physical demands (OR 4.38). The fourfold increased risk of reporting high physical demands in these two working populations may be indicative of cumulative disadvantage over a long career, the deleterious effects of chronic exposure to hard manual labour or physical exertion, or age-related reductions in strength (Keller and Engelhardt 2013; Sundstrup et al. 2016; Ervasti et al. 2019).

Assessing how CHCs affect physical demands is vital because physical capacity decreases as individuals age. Adults can observe annual muscle decrements of up to 2% starting at age 30, with approximately 30% of muscle strength lost by age 50 (Sundstrup et al. 2016). One study reported muscle strength decline of over 40% in adults over the age of 40, implying an increased rate of deceleration (Keller and Engelhardt 2013). The loss of muscle mass and strength, which is independently associated with chronic illness, makes physical work such as carrying heavy loads, prolonged standing, and frequent bending challenging and, in some cases, unmanageable for older workers.

Health-care workers also had the highest risk of emotional demands in our analysis. The daily emotional toll of caring for gravely sick patients, seeing the death of those they have cared for, and being exposed to hostile patients can negatively impact workers in the health sector (Franzosa et al. 2018). Similarly, the education and welfare sectors also had a twofold increased risk of emotional demands. Studies have shown that student behaviour can be emotionally demanding for teachers (Bakker et al. 2007; Chang 2009; Lee and van Vlack 2018), and welfare workers often feel emotionally extended due to interactions with clients, with child welfare workers experiences greater emotional demands and exhaustion (Kim 2011; Lizano and Mor Barak 2012).

Emotionally demanding work which involves direct client, customer or patient contact, exposure to their behavioural characteristics and potentially traumatic events (de Jonge et al. 2010) can invoke strong feelings such as sorrow, anger, desperation, and frustration. Chronic exposure to emotionally stressful work is associated with burnout, job dissatisfaction, absenteeism, disability pension, and presenteeism (Scheibe et al. 2015; Salvagioni et al. 2017). Our findings contribute to understanding the differential association between poor chronic health and perceived general stress, physical and emotional demands in older workers and may help inform conversations and decisions about appropriate allocation of resources to intervene and mitigate associated negative effects on general well-being and quality of life.

Our study has several limitations. First, use of cross-sectional data did not allow us to establish causality. Second, due to the cross-sectional nature of the data, we were unable to control for time-related employment status, to assess HWSE. Third, our response rate of 44% was low and may have introduced non-response bias to our study. This non-response bias may underestimate the associations between chronic disease and work-related stress, as previous studies have indicated that relative to individuals with good health and life experiences, individuals with poor health are less likely to participate in surveys (Cheung et al. 2017). We also readily acknowledge that our use of a single item response for the outcomes is a limitation, in particular as there are some validated stress measures that have been used in the literature. Further emotional demands are individually variant and subjective, which may increase the likelihood of random error or systematic bias (Mutambudzi and Javed 2016). Our study, however, is strengthened by the use of a large dataset of Dutch adults approaching the age of retirement.

The findings we present here cannot not be taken to translate straightforwardly as describing the bidirectional relationship between CHCs and work stress. Instead, we believe that these results may be useful as one contributing element in understanding how stress is an outcome of poor health. Taken together with other studies, this work may inform optimal self- and clinical management interventions for older workers, to ensure they are healthy and able to remain productive.

The proportion of older workers with CHCs will continue to increase over the years. Understanding how their health may increase susceptibility to stress and subsequent health outcomes is therefore important. Continued research in this area is of great importance in order to gather evidence that will allow for effective workplace and public health interventions. Future research needs to take into account disease severity, longitudinal employment history to account for HWSE, and disease self-management as these are likely to impact work ability and mediate the relationship between CHCs and work stress.
